# Supplementary material for: Poly(ADP-ribose) polymerase 1 accelerates vascular calcification by upregulating Runx2
Source: Nat Commun. 2019 Mar 13;10:1203. doi: 10.1038/s41467-019-09174-1 (PMC6416341; doi:10.1038/s41467-019-09174-1)
Supplement: Supplementary file 1 — Supplementary Information [file 41467_2019_9174_MOESM1_ESM.docx]

**SUPPLEMENTARY INFORMATION**

**Poly(ADP-ribose) polymerase1 accelerates vascular calcification by upregulating Runx2**

*Cheng Wang et al.*


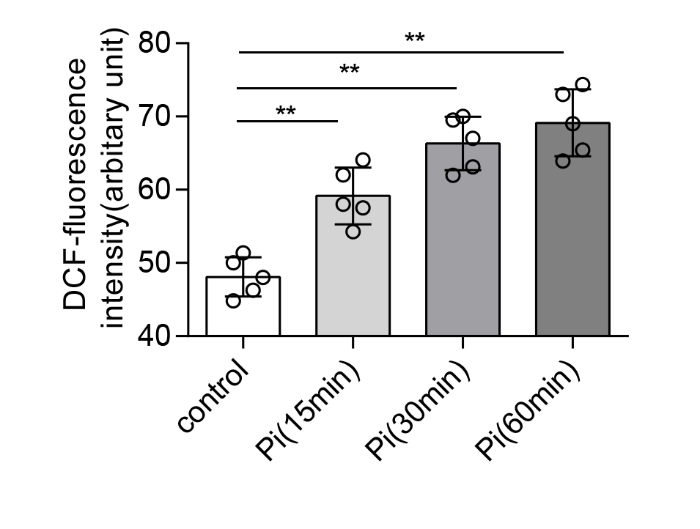


**Supplementary Figure 1. Oxidative stress is enhanced under high Pi stimulus.** Rat VSMCs were treated with high Pi for 0, 15, 30 and 60 minutes. The oxidative stress was detected by DCF-fluorescence. (n=5 per group). Statistical significance was assessed using one-way ANOVA followed by for multiple comparison.^**^*P* < 0.01. All values are means ± S.D..


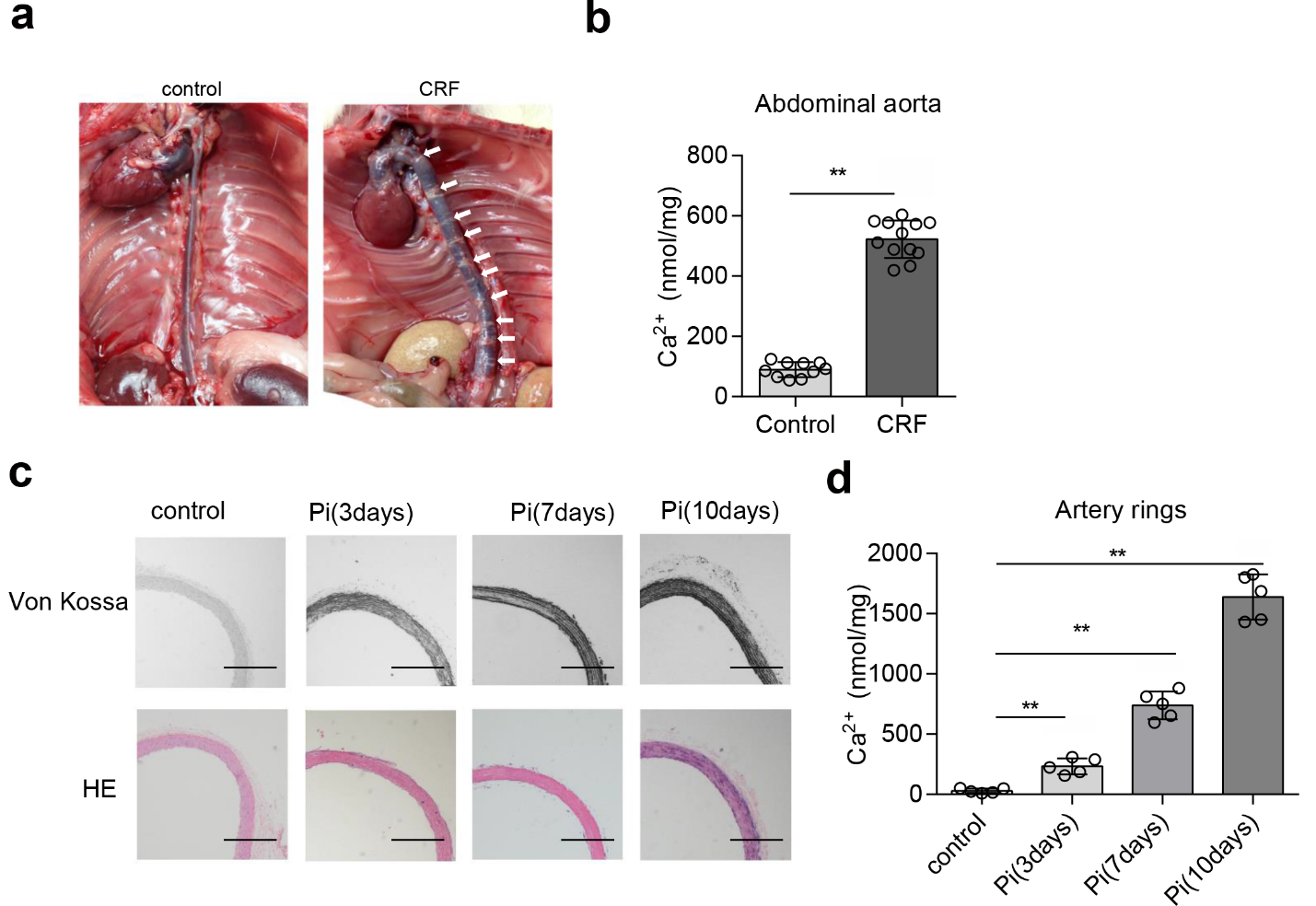


**Supplementary Figure 2. Vascular calcification is successfully induced in calcified arteries. a** and **b**, Wistar rats were fed an 0.75% adenine diet for 6 weeks. Mineral nodules were detected by morphology (**a**). The calcium content in arteries was calculated **(b)**. (n=10-12 per group).**c** and **d**, Rat aortic rings were treated with high Pi (10mM β-glycerophosphate) for indicated days (0, 3, 7 and 10 days). Von Kossa and HE staining were assayed (**c**). The calcium in rings were quantified (**d**). (n=5 per group). Scale bar, 200µm .Statistical significance was assessed using one-way ANOVA for multiple comparison and two-tailed t-tests for two group and is presented as follows: ** *P* < 0.01 and ^##^ *P* < 0.01. All values are means ± S.D.

**
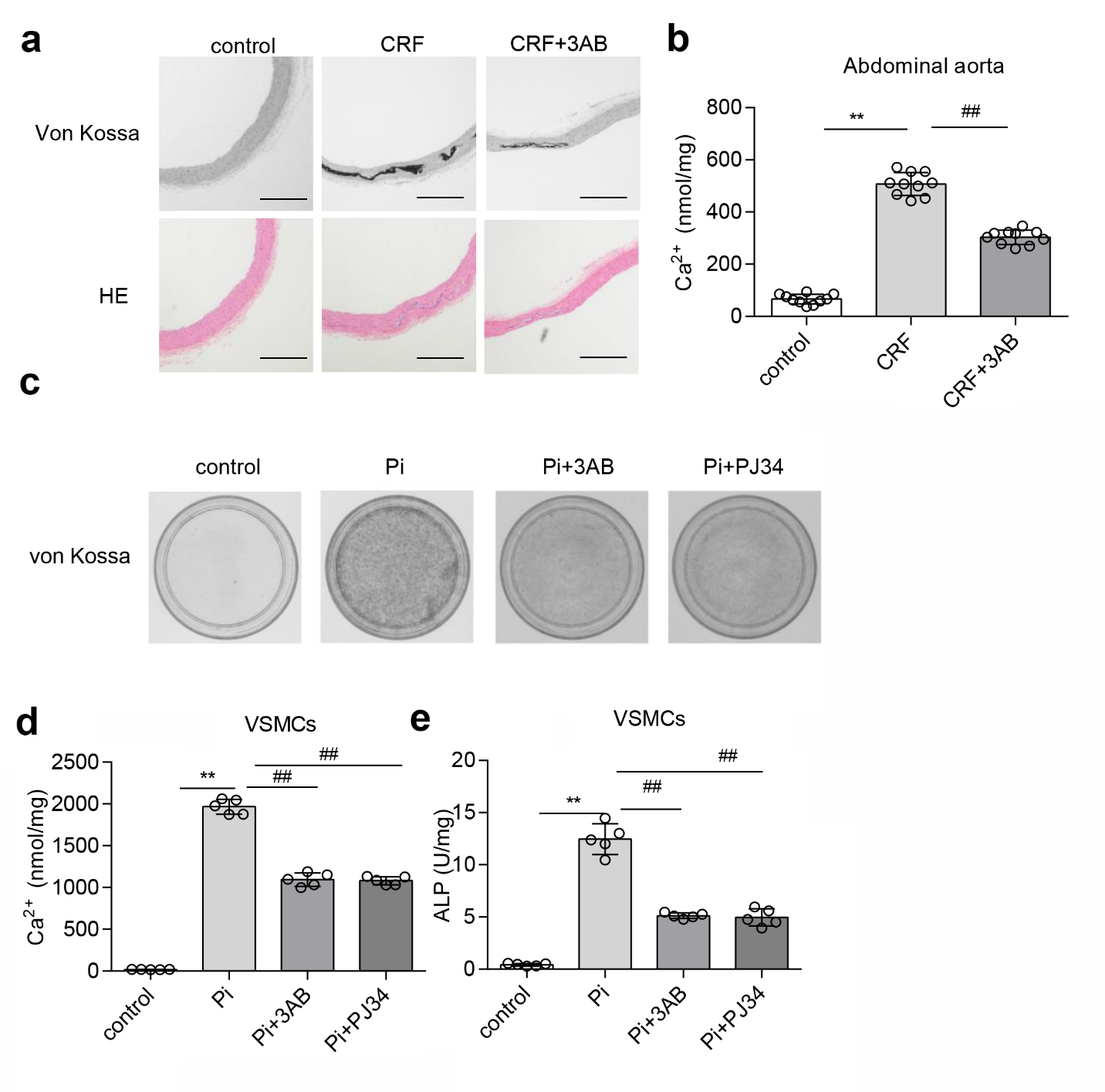
**

**Supplementary Figure 3**. **PARP1 inhibitor 3AB attenuates vascular calcification**. **a** and **b**, CRF rats were randomly received intraperitoneal injection of 3AB (10mg/kg/d) or vehicle once a day. Aortas were stained for mineralization by von Kossa and H&E staining (**a**), and the quantitative analysis of calcium deposition were detected (**b**). (n=10-12 per group). **c**-**e**, Rat primary VSMCs were treated with 3AB (10mM) and incubated with osteogenic medium for 14 days. VSMCs were stained for mineralization by Alizarin red S (**c**), and the quantitative analysis of calcium content (**d**) and ALP (**e**) were detected. (n=5 per group). Scale bar, 200μm. Statistical significance was assessed using one-way ANOVA for multiple comparison and is presented as follows: ** *P* < 0.01 and ^##^ *P* < 0.01. All values are means ± S.D.

**
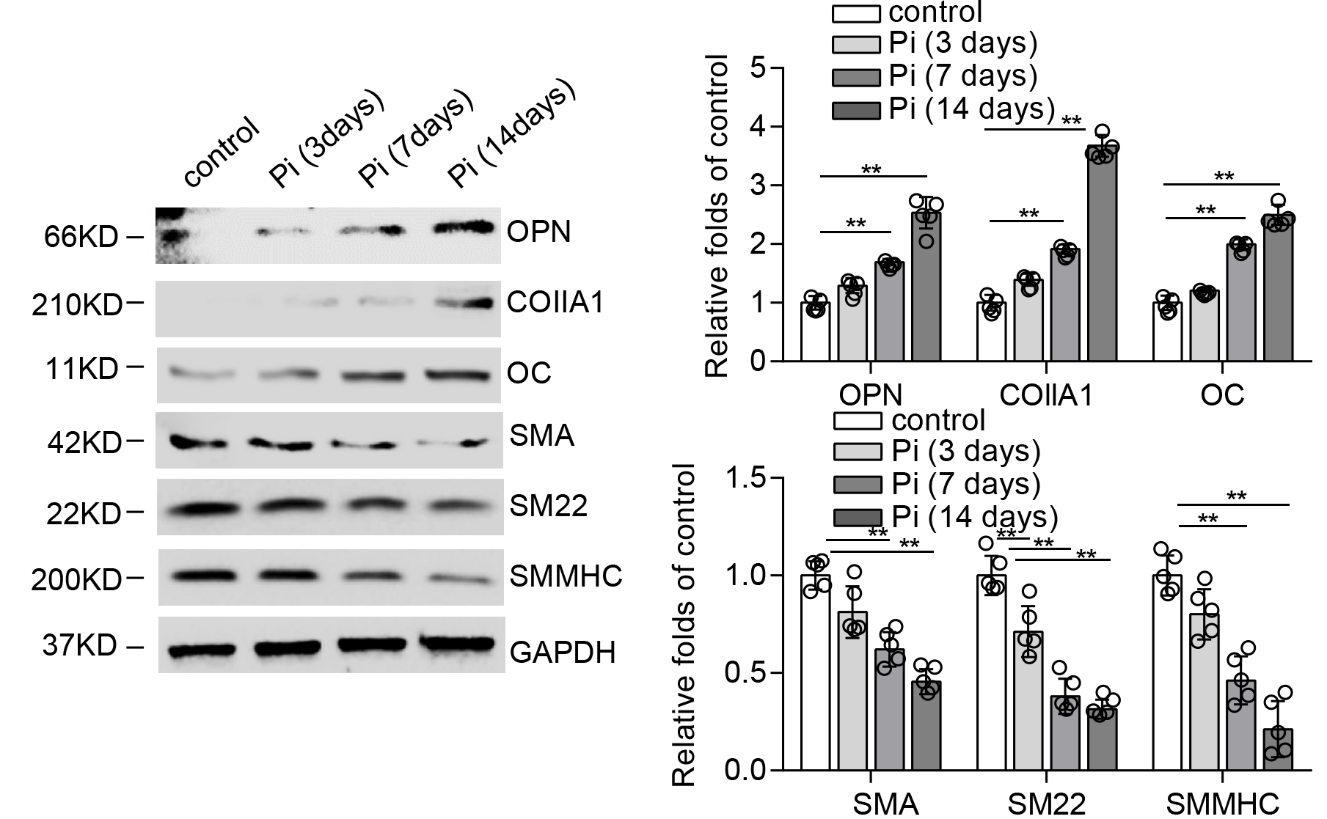
**

**Supplementary Figure 4**. **High Pi induces VSMC osteogenic transdifferentiation**. Representative western blot analysis of osteogenic markers (OPN, ColIA1 and OC) and smooth muscle lineage markers (SMA, SM22 and SMMHC) expressions in rVSMCs treated with high Pi for 14 days. Statistical significance was assessed using one-way ANOVA followed for multiple comparison, ^**^ *P* < 0.01. All values are means ± S.D.


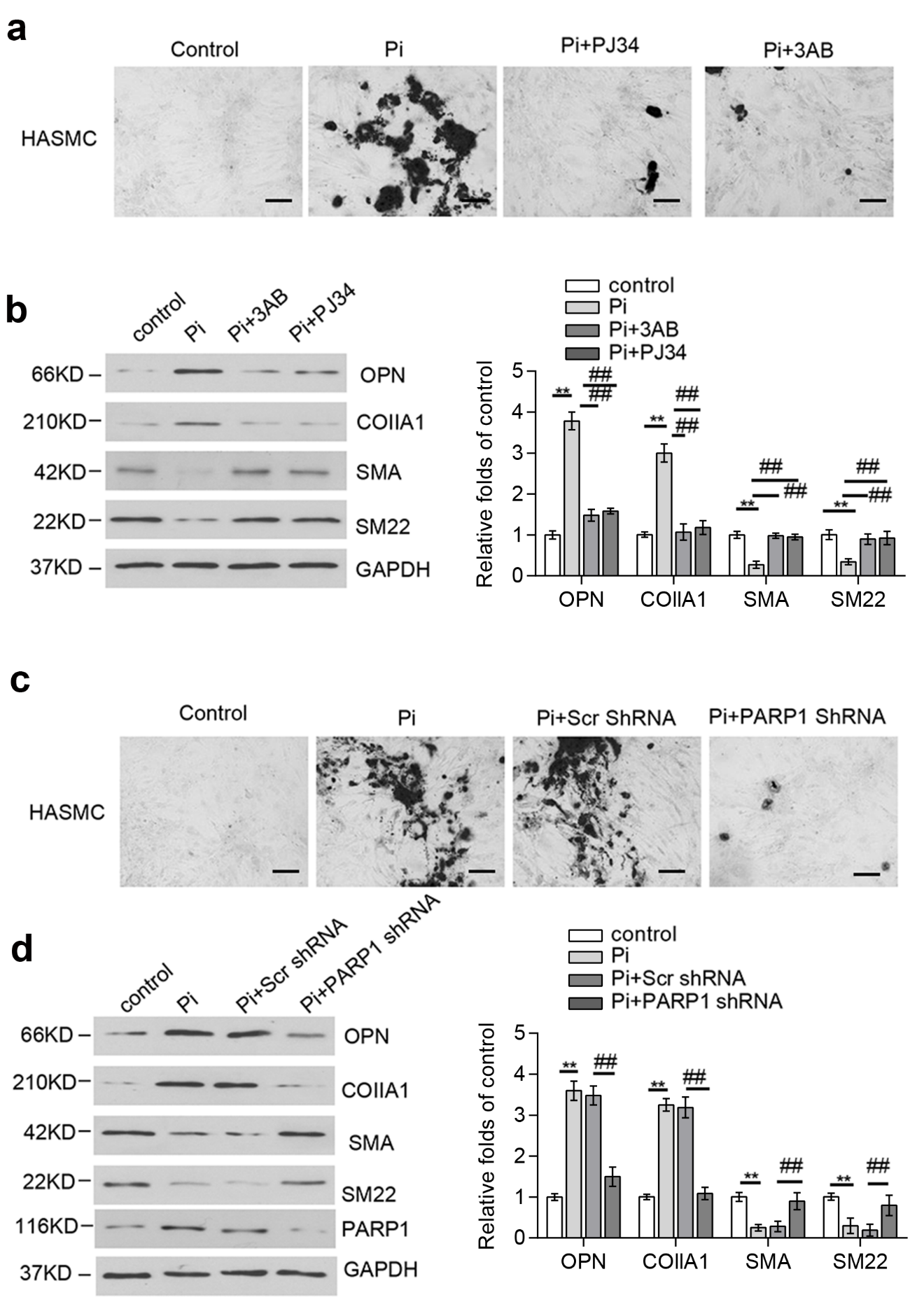


**Supplementary Figure 5**. **PARP1 inhibition or deficiency suppresses HASMC osteogenic transition and calcification.** **a** and **b**, Human aortic SMCs (HASMCs) were incubated with osteogenic medium with 3AB or PJ34 for 14 days. VSMCs were stained for mineralization by Alizarin red S (**a**), and the expressions of osteogenic markers (OPN and ColIA1) and smooth muscle lineage markers (SMA and SM22) were detected by western blot assay (**b**). **c** and **d**, HASMCs were pre-infected with Scrambled or PARP1 shRNA adenovirus and then exposed to osteogenic medium for 14 days. VSMCs were stained for mineralization by Alizarin red S (**c**). The osteogenic markers (OPN and ColIA1) and smooth muscle lineage markers (SMA and SM22) were detected by western blot assay (**d**). (n=5 per group). Scale bar = 20μm. Statistical significance was assessed using one-way ANOVA for multiple comparison and is presented as follows: ^**^ *P* < 0.01 and ^##^ *P* < 0.01. All values are means ± S.D. Source data are provided as a Source Data file.

**
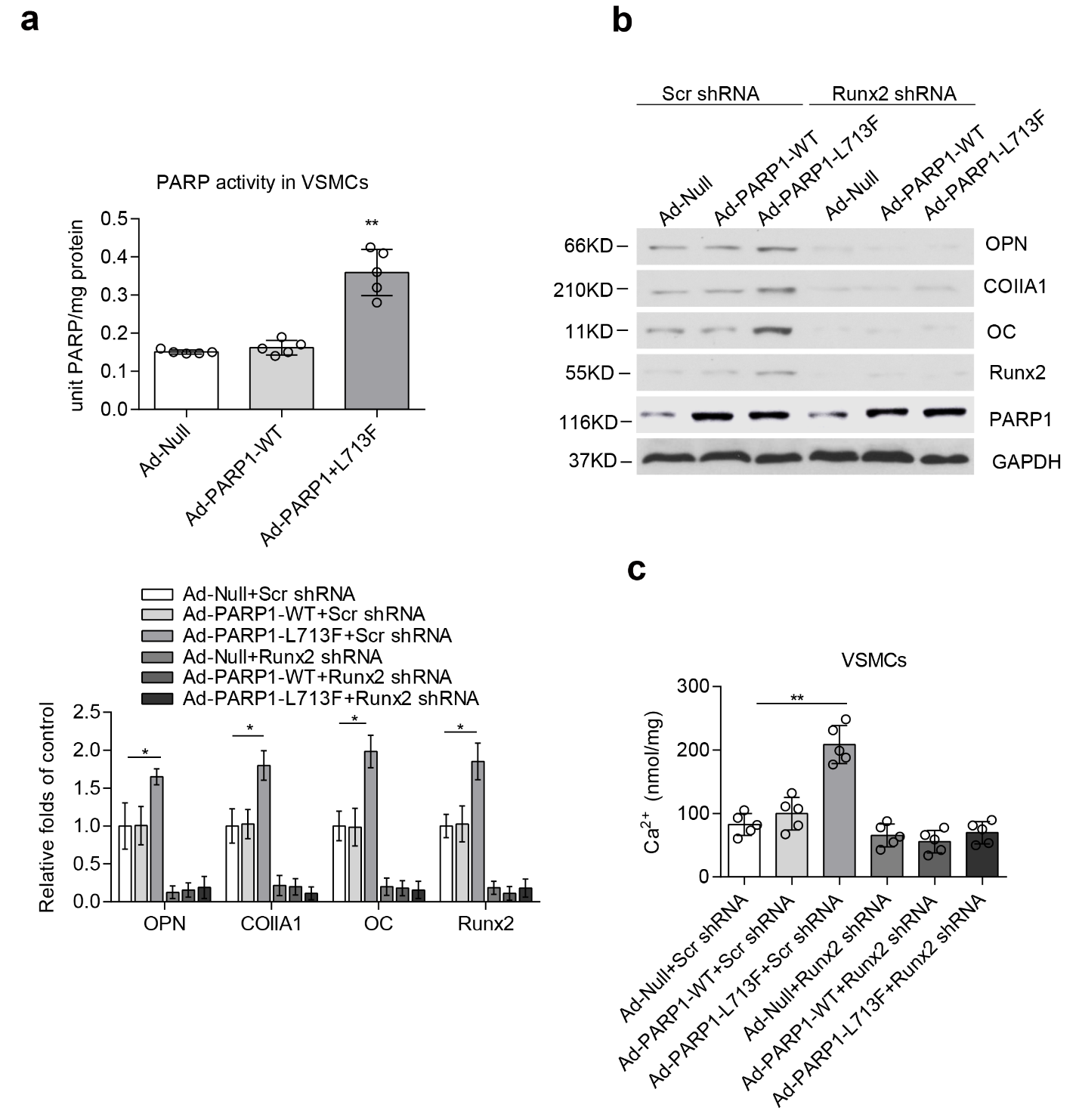
Supplementary Figure 6**. **Runx2 mediates the effects of activated PARP1 on VSMC osteogenic transition.** Rat VSMCs were infected with Ad-Null, Ad-PARP1-WT or Ad-PARP1-L713F，together with Scr shRNA or Runx2 shRNA, and then incubated with normal media for 3 days. **a**, The PARP activity in Ad-Null, Ad-PARP1-WT and Ad-PARP1-L713F infected VSMCs were assayed. The osteogenic markers (OPN, ColIA1, OC and Runx2) (**b**), and calcium content (**c**) were determined. (n=5 per group). Statistical significance was assessed using one-way ANOVA for multiple comparison, ^*^ *P* < 0.05 and ^**^ *P* < 0.01. All values are means ± S.D. Source data are provided as a Source Data file.

**
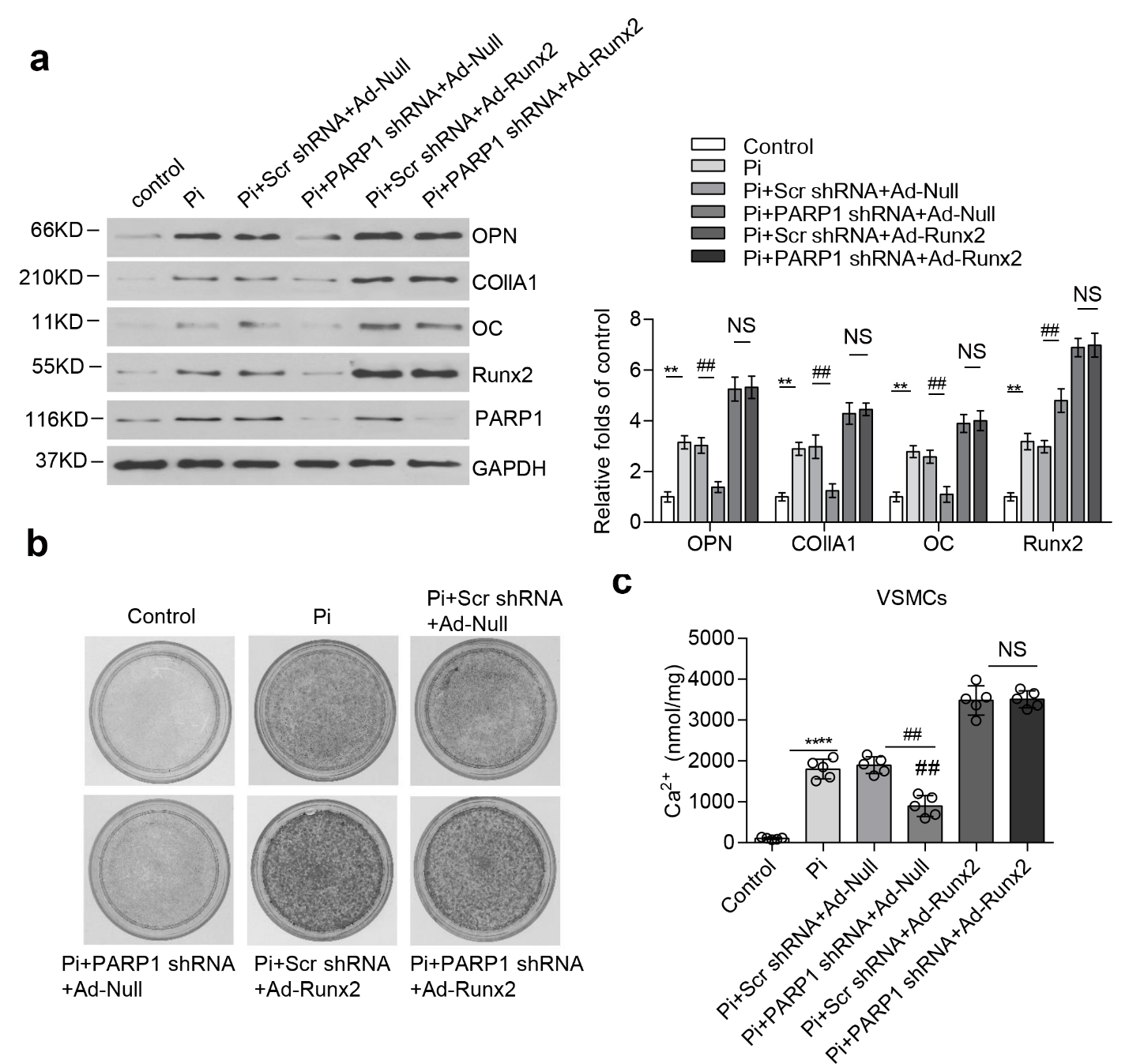
Supplementary Figure 7**. **Runx2 overexpression antagonizes the protective effects of PARP1 deficiency on VSMC calcification.** VSMCs were infected with Scr shRNA or PARP1 shRNA, together with Ad-Null or Ad-Runx2 for 48 hours and then incubated with osteogenic media for 14 days. The osteogenic markers expression (OPN, ColIA1, OC and Runx2) and relative quantification (**a**), Alizarin red S staining (**b**), and the calcium content (**c**) in calcified VSMCs were determined. (n=5 per group). Statistical significance was assessed using one-way ANOVA for multiple comparison and is presented as follows: NS: no significance, ^**^ *P* < 0.01 and ^##^ *P* < 0.01. All values are means ± S.D. Source data are provided as a Source Data file.

**
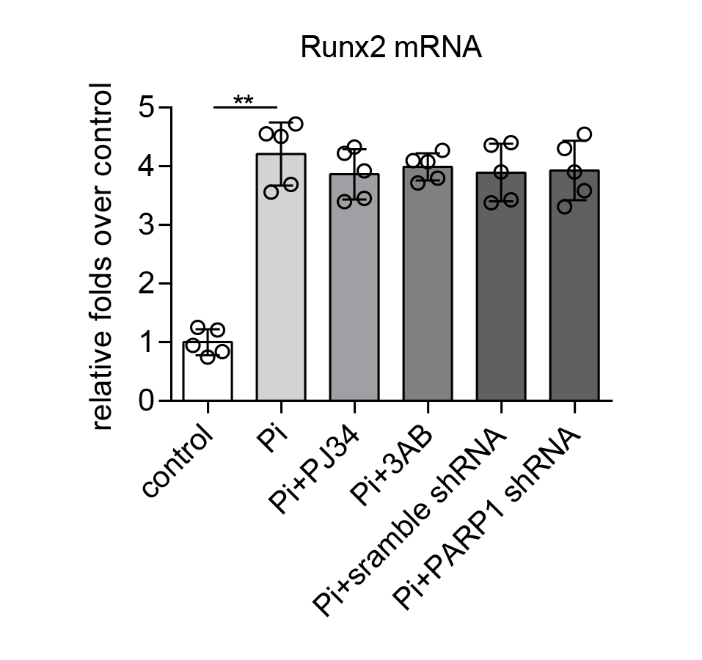
**

**Supplementary Figure 8**. **PARP1 has no effects on the mRNA level of *Runx2***. Rat VSMCs was treated with PARP1 inhibitors or PARP1 shRNA, and then exposed to osteogenic media. The mRNA level of *Runx2* was determined by qRT-PCR. Statistical significance was assessed using one-way ANOVA for multiple comparison. ^**^ *P* < 0.01. All values are means ± S.D.

**
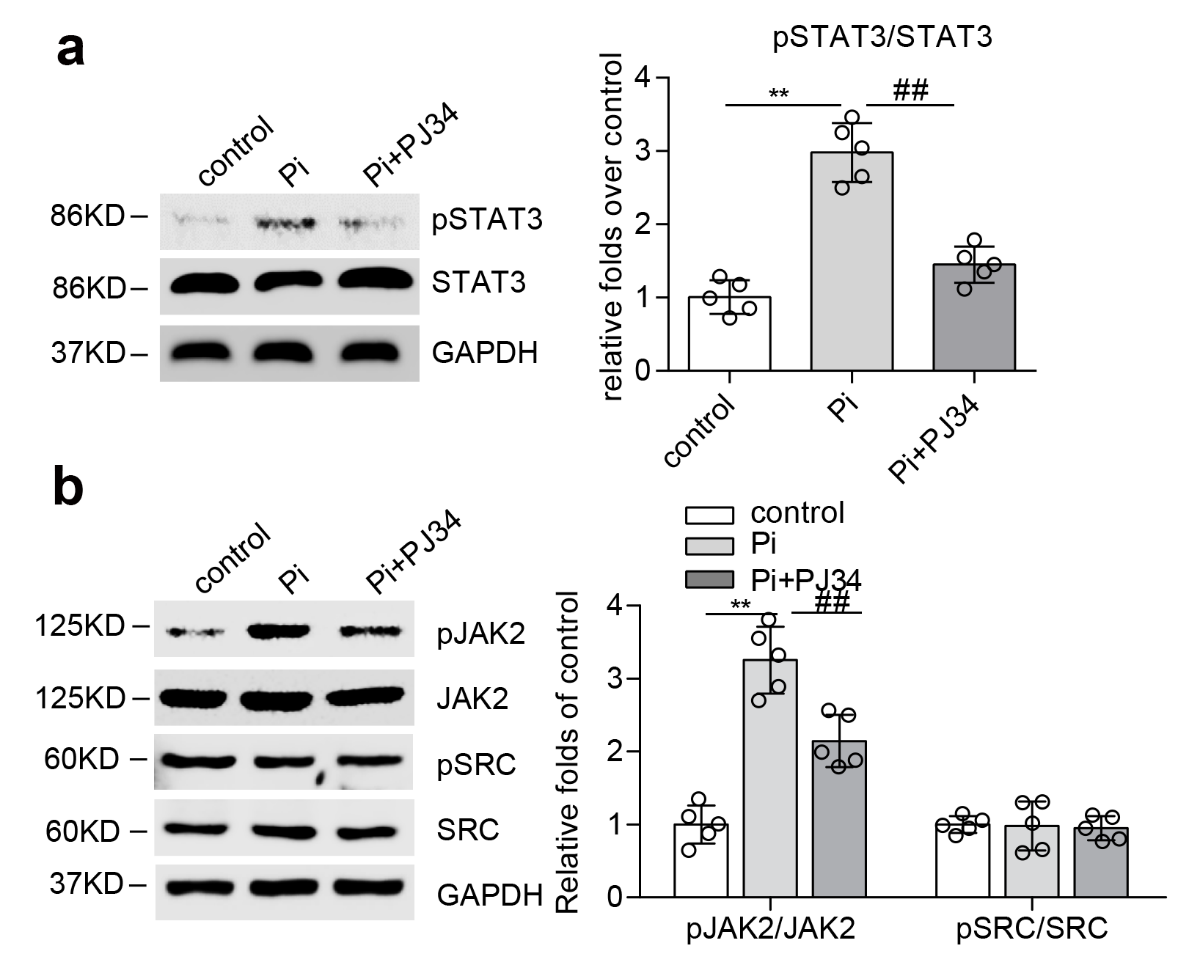
**

**Supplementary Figure 9**. **PARP inhibitor PJ34 decreases JAK2/STAT3 activation.** Rat VSMCs were exposed to high Pi for 3 days, in the absence or presence of PJ34 (10μM). The levels of pSTAT3 (**a**), pJAK2 and pSrc (**b**) and their total proteins were determined by western blot. (n=5 per group). Statistical significance was assessed using one-way ANOVA for multiple comparison and is presented as follows: ^**^ *P* < 0.01 and ^##^ *P* < 0.01. All values are means ± S.D.


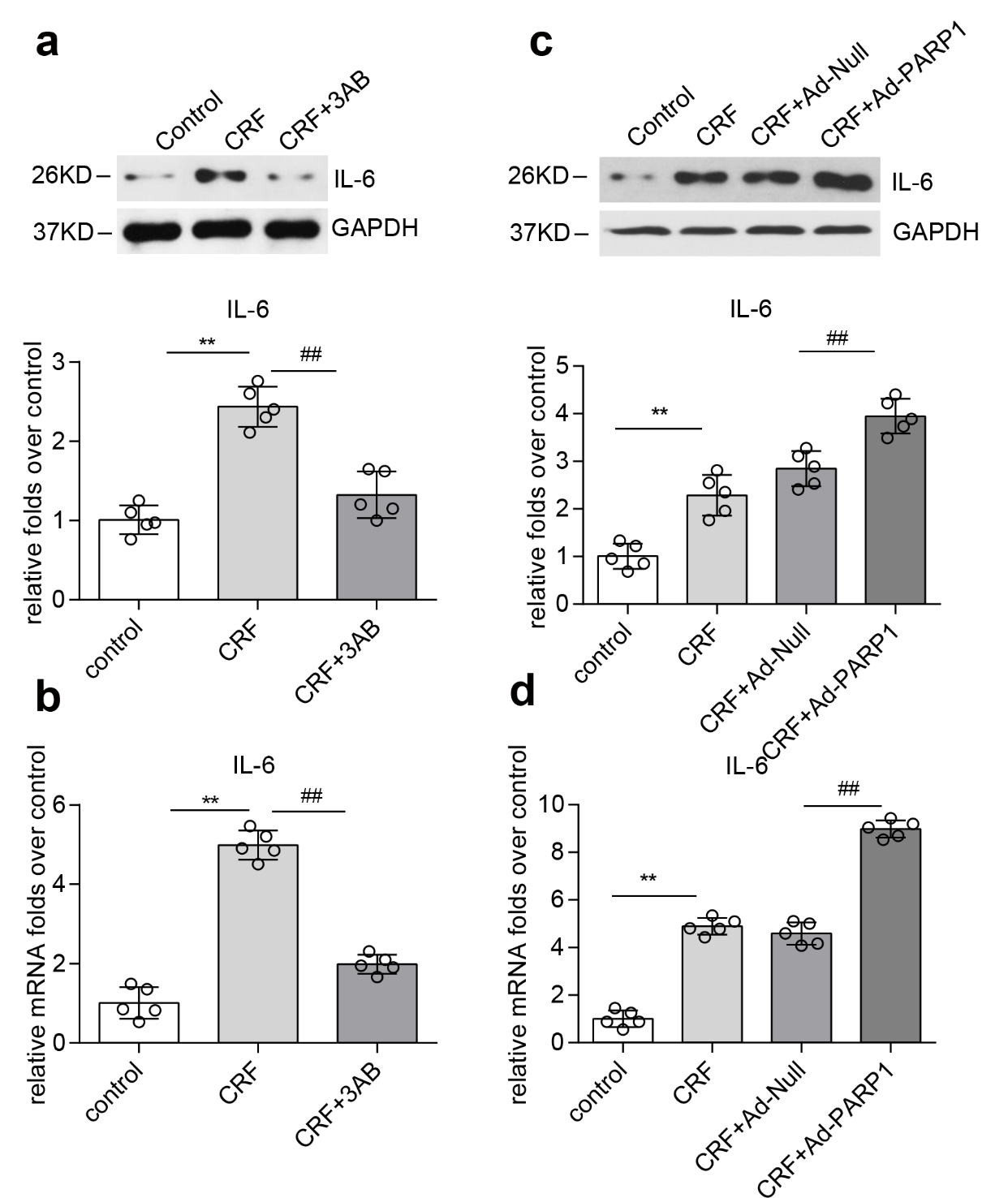


**Supplementary Figure 10**. **PARP1 regulates arterial IL-6 expression in CRF rats.** CRF rats were randomly received intraperitoneal injection of 3AB (10mg/kg/d) or vehicle once a day for the latter three weeks. The protein level of IL-6 in abdominal aortas was determined by western blot assay (**a**, **c**). The mRNA level of IL-6 was determined by qRT-PCR (**b**, **d**). (n=5 per group). Statistical significance was assessed using one-way ANOVA for multiple comparison and is presented as follows: ^**^ *P* < 0.01 and ^##^ *P* < 0.01. All values are means ± S.D.

**
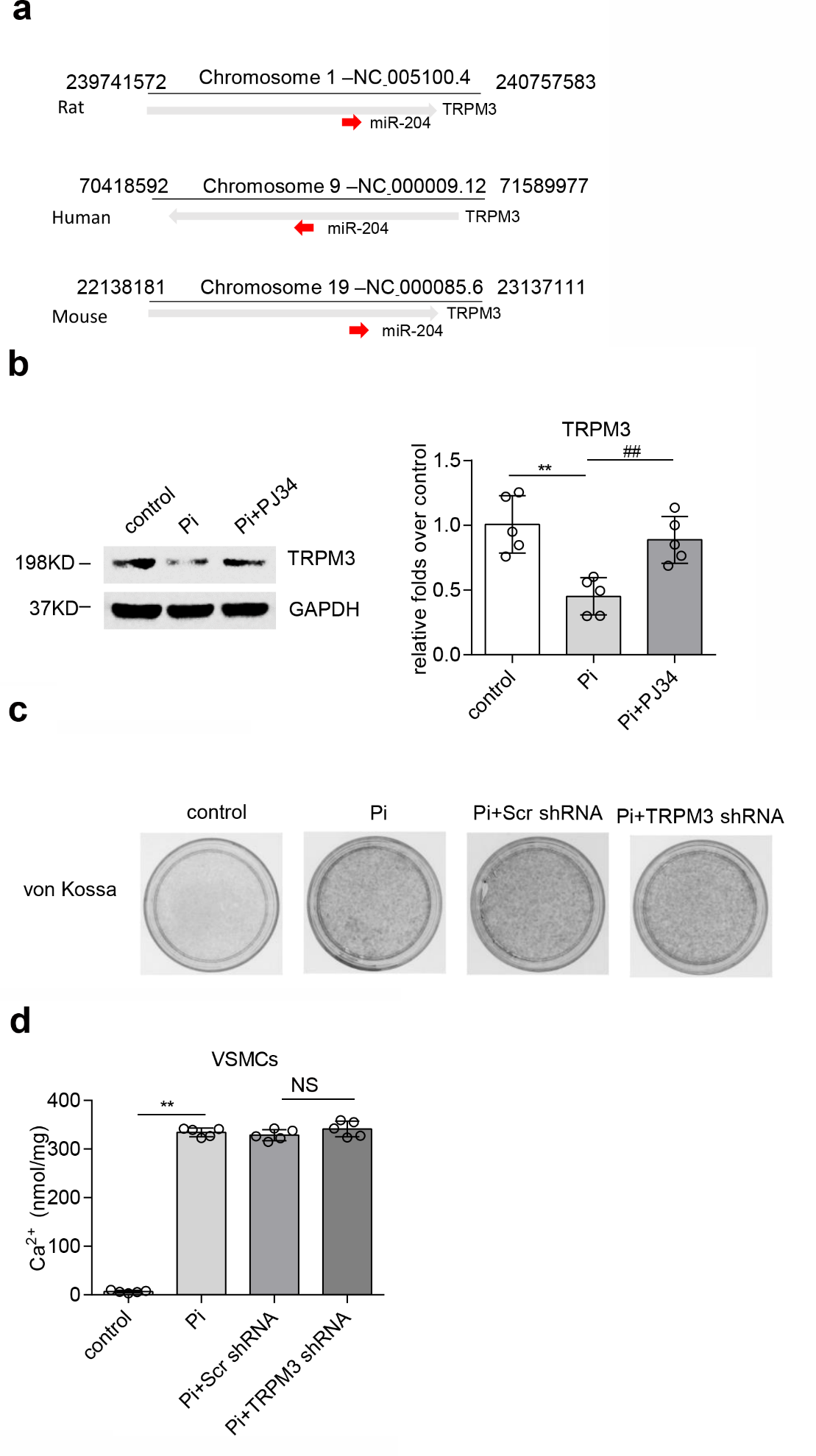
**

**Supplementary Figure 11. TRPM3 is not involved in PARP1-mediated VSMCs calcification.**  **a**, Cartoon depiction of the miR-204/TRPM3 locus. The gene of TRPM3 were marked as gray box. MiR-204 region was marked as red. **b**, rVSMCs were treated with high Pi or together with PJ34, and then subjected to western blot for TRPM3 level. **c** and **d**, VSMCs were infected with Scr or TRPM3 shRNA, and incubated in osteogenic media for 14 days. Alizarin red S staining was performed (**c**) and the calcium content was quantified (**d**). (n=5 per group). Statistical significance was assessed using one-way ANOVA for multiple comparison and is presented as follows: NS: no significance, ^**^ *P* < 0.01 and ^##^ *P* < 0.01. All values are means ± S.D.


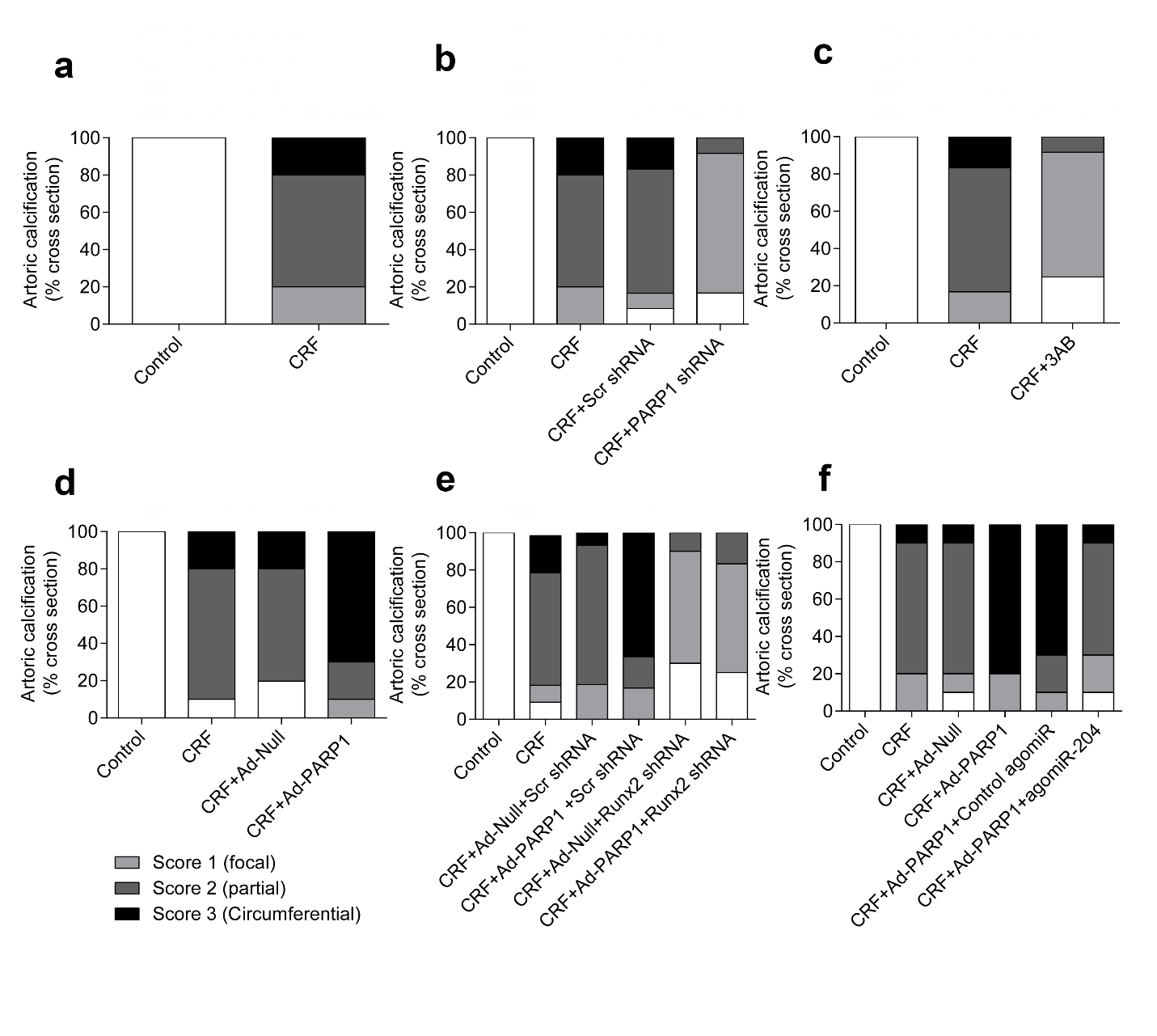


**Supplementary Figure 12. The quantification of relative von Kossa staining.** The extent of aortic calcification was evaluated based on a semiquantitative scoring of von Kossa-stained aortic sections. **a**, The quantification of calcification in control and CRF group. **b**, The quantification of calcification in control, CRF, CRF+Ad-Scr shRNA and CRF+Ad-PARP1 shRNA groups. **c**, The quantification of calcification in control, CRF, CRF+3AB groups. **d**, The quantification of calcification in control, CRF, CRF+Ad-Null and CRF +Ad-PARP1 groups. **e**, The quantification of calcification in control, CRF, CRF + Ad-Null+ Ad-Scr shRNA, CRF+Ad-PARP1+Ad-Scr shRNA, CRF+Ad-Null+ Ad-Runx2 shRNA and CRF+Ad-PARP1+Ad-Runx2 shRNA groups. **f**, The quantification of calcification in control, CRF, CRF+Ad-Null, CRF+Ad-PARP1, CRF+Ad-PARP1+Control agomir and CRF+Ad-PARP1+agomiR-204 groups.


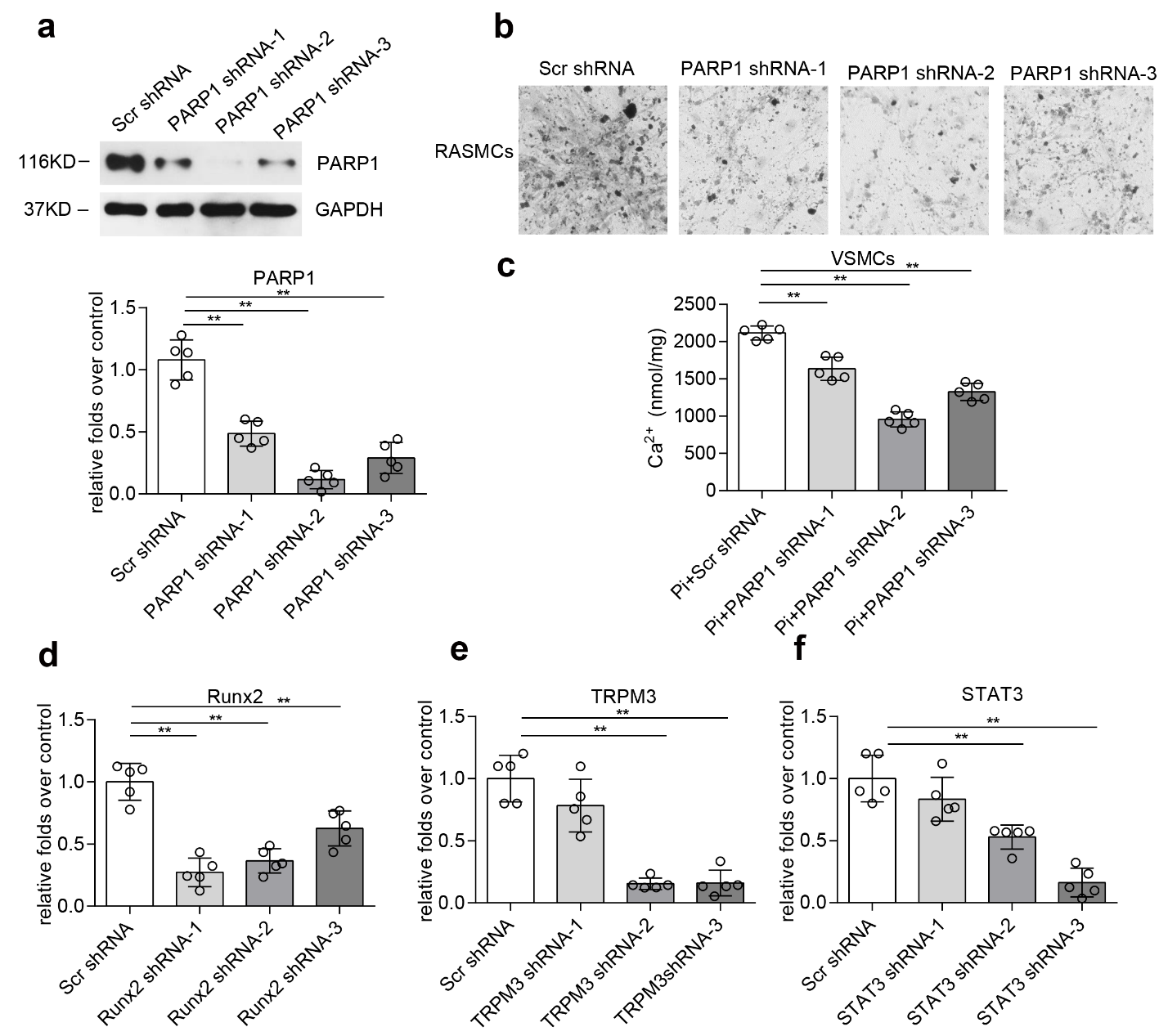


**Supplementary Figure 13**. **The efficiency of PARP1, Runx2, TRPM3 and STAT3 shRNA**. **a**-**c**, VSMCs were infected with adenovirus encoding three different lines of PARP1 shRNA for 48 hours, and then exposed to osteogenic media for 14 days. The protein level of PARP1 was determined by western blot assay (**a**). VSMCs were stained for mineralization by Alizarin red S (**b**), and the quantitative analysis of calcium content were detected (**c**). **d**-**f**, VSMCs were separately infected with Scr shRNA , three different lines of Runx2 shRNA, TRPM3 shRNA and STAT3 shRNA for 48 hours, and then the mRNA level of *Runx2* (**d**), *Trpm3*(**e**) and *Stat3* (**f**) were determined by qRT-PCR. (n=5 per group). Statistical significance was assessed using one-way ANOVA for multiple comparison, ^**^ *P* < 0.01. All values are means ± S.D.


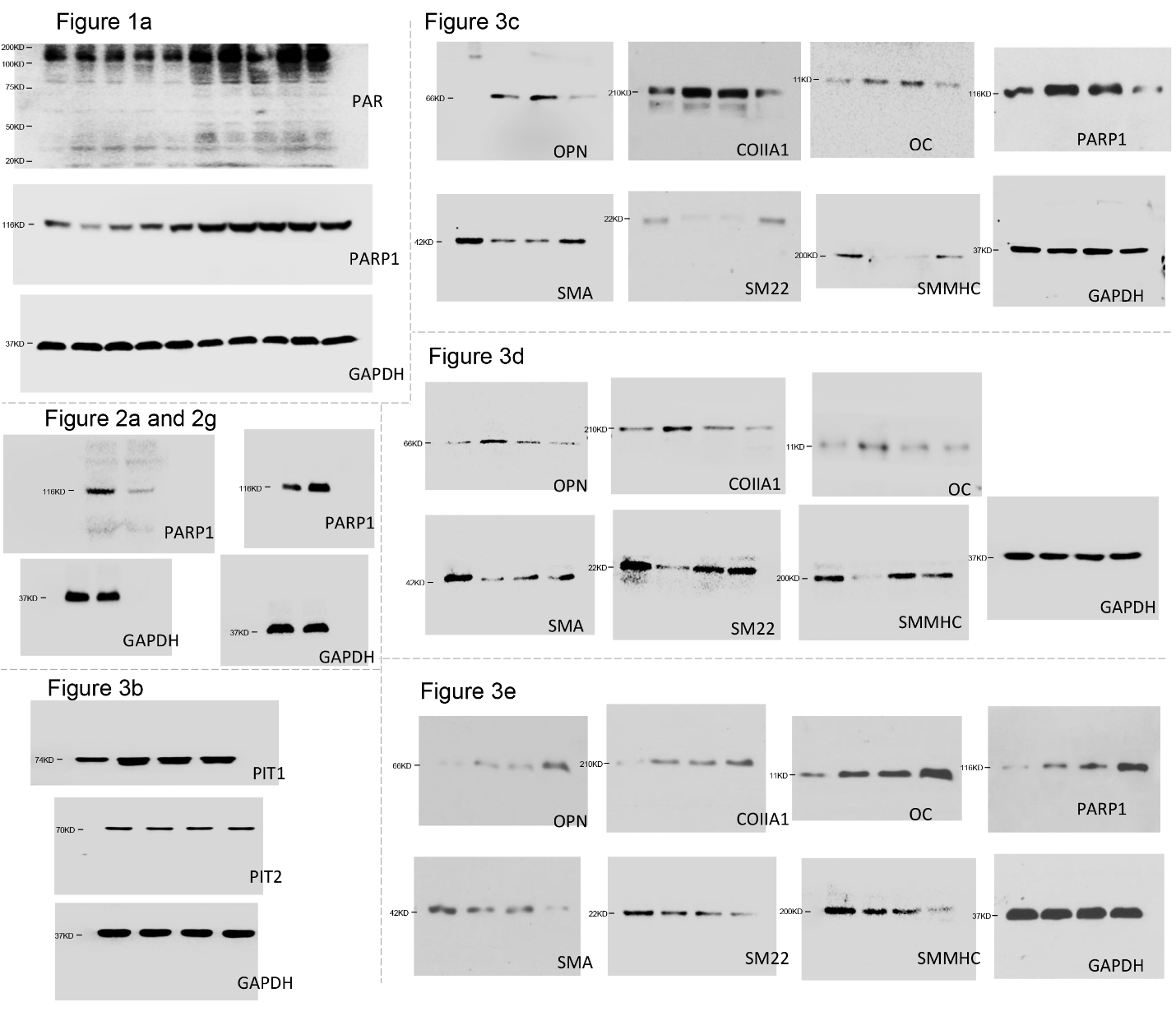


**Supplementary Figure 14. Uncropped scans of the western blots of Figure1-3**.


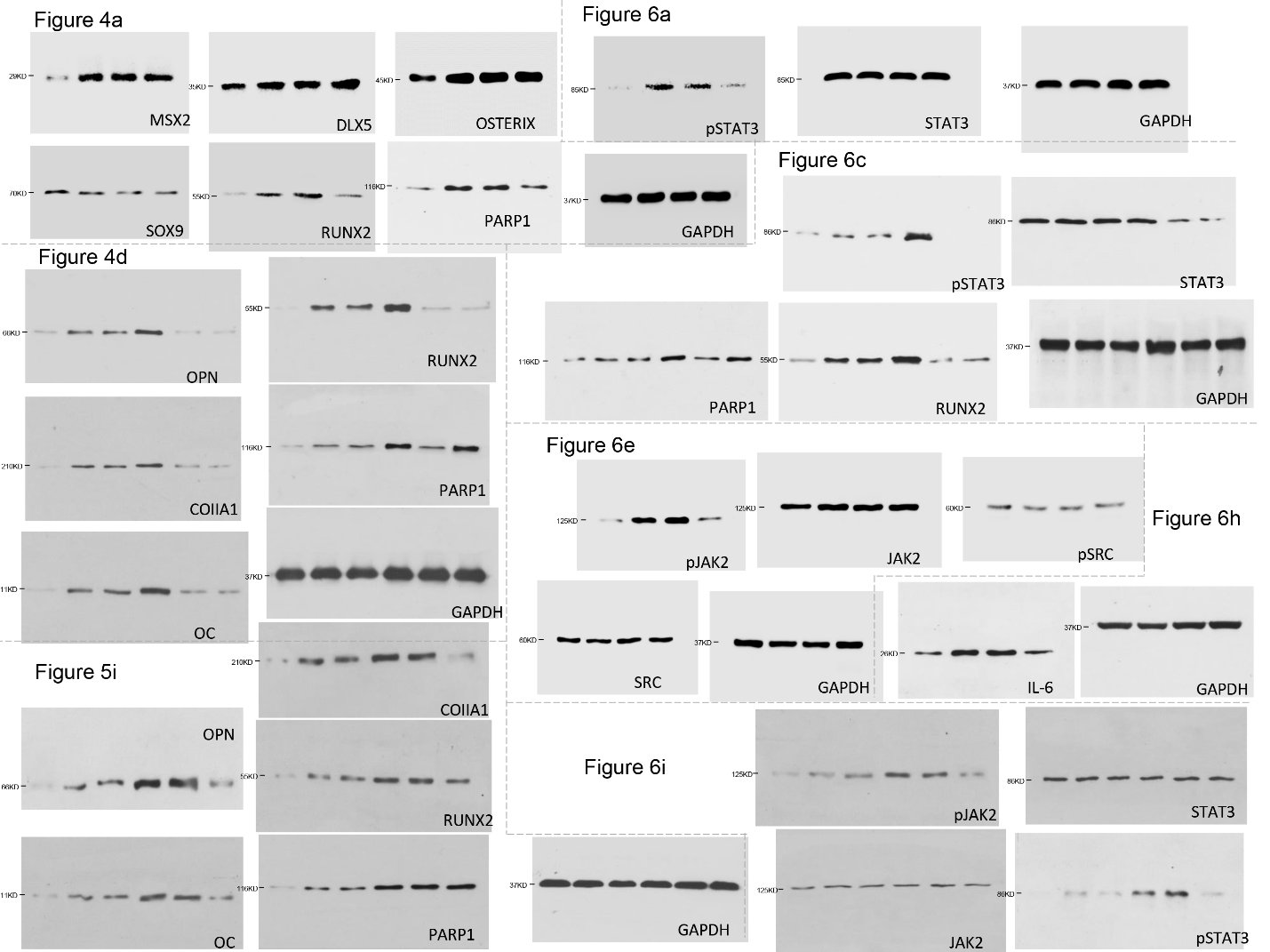


**Supplementary Figure 15. Uncropped scans of the western blots of Figure 4-6**.


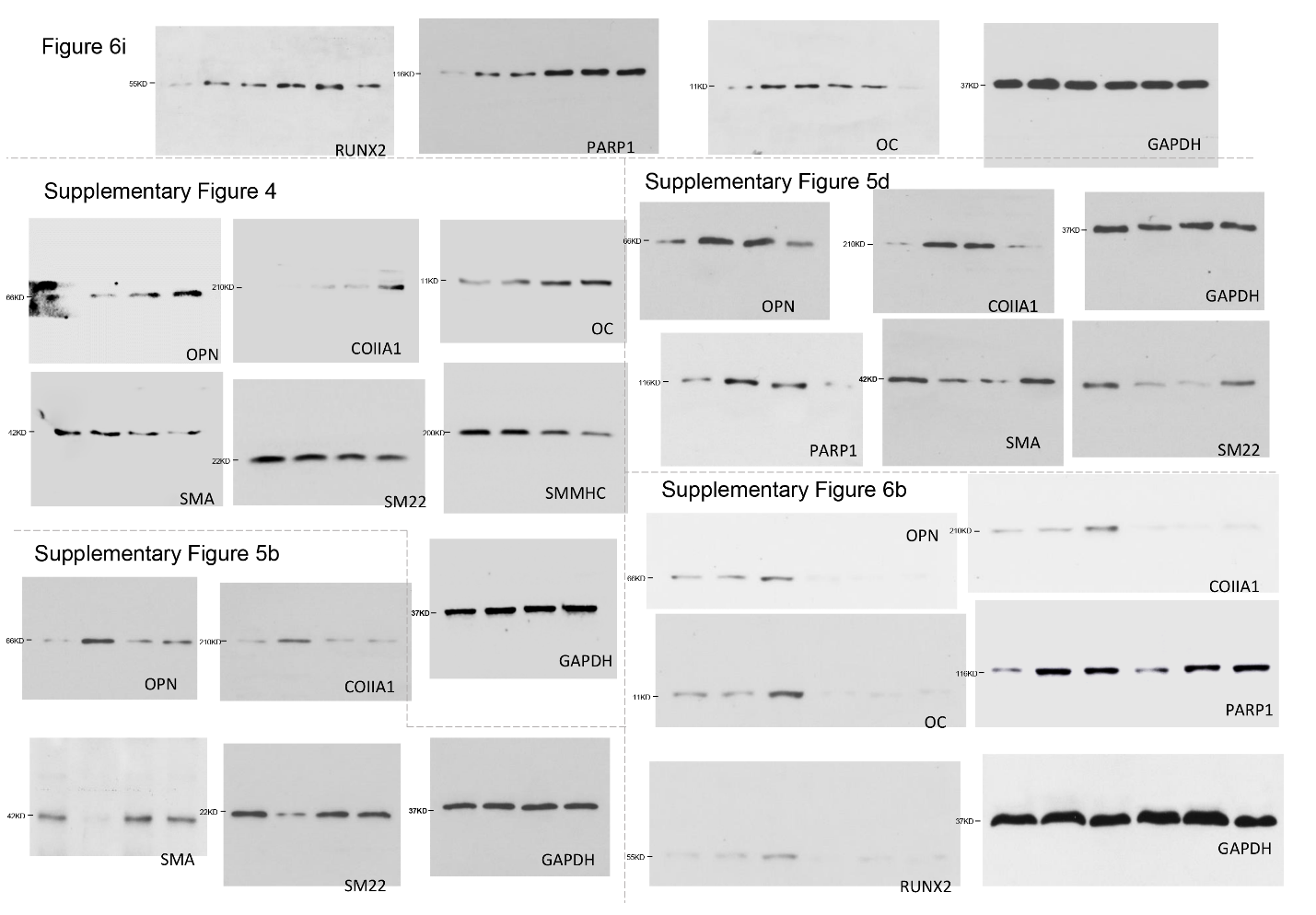


**Supplementary Figure 16. Uncropped scans of the western blots of Figure 6 and Supplementary Figures**.


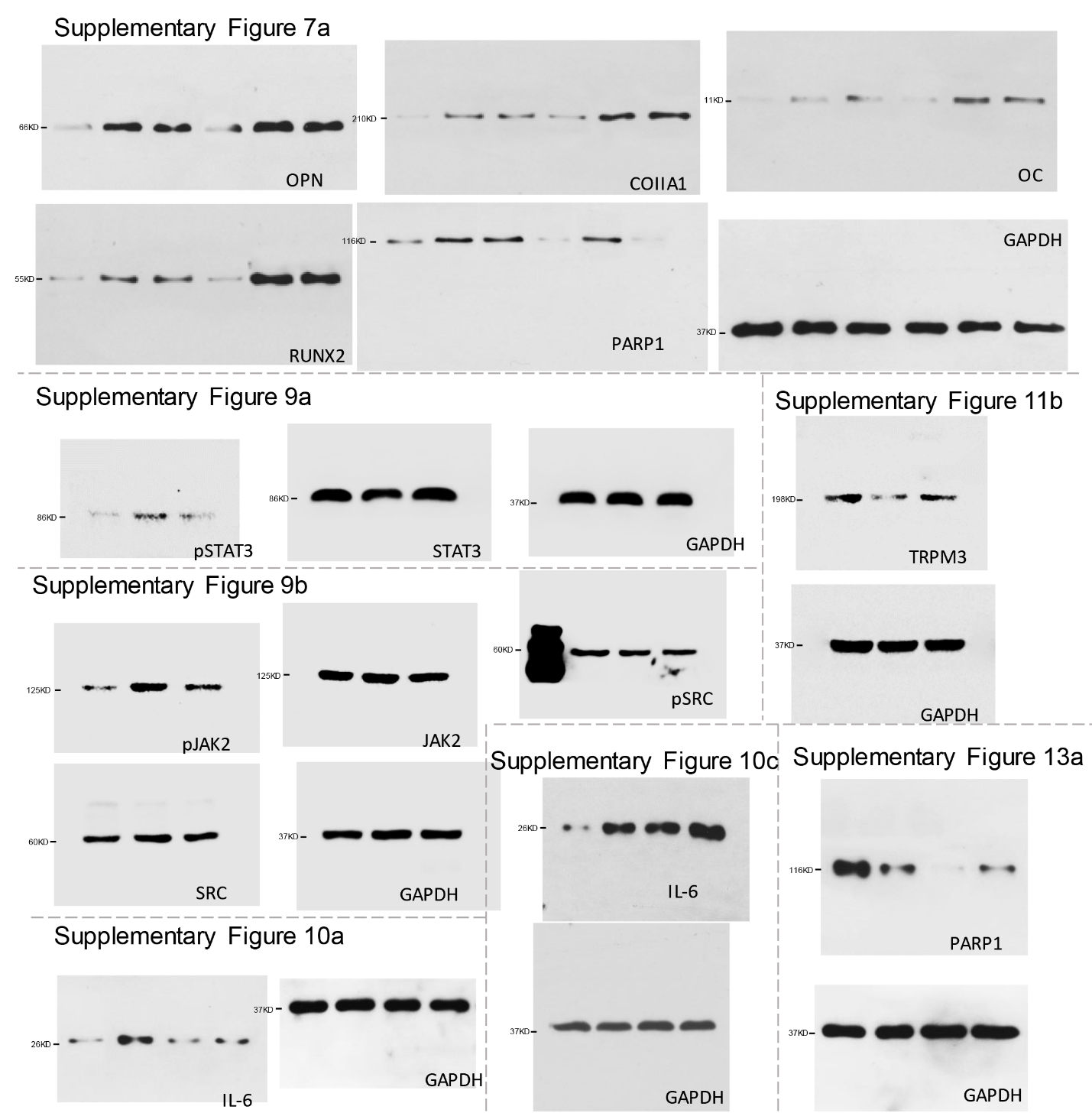


**Supplementary Figure 17. Uncropped scans of the western blots of Supplementary Figures**.

**Supplementary Table 1. Serum biochemical parameters and body weight of CRF rats with PARP inhibitor 3AB.**

| **Parameters** | control | CRF | CRF+3AB |
| --- | --- | --- | --- |
| Body weight (g) | 457.32±30.29 | 257.33±23.01^**^ | 254.11±16.79 |
| Phosphorus(mM) | 2.45±0.15 | 4.55±0.32^**^ | 4.31±0.23 |
| Calcium (mM) | 2.39±0.27 | 2.45±0.24 | 2.46±0.29 |
| BUN (mM) | 6.43±1.38 | 80.80±5.67^**^ | 77.40±5.60 |
| Cr(µM) | 63.77±14.33 | 295.97±35.44^**^ | 304.11±25.28 |

CRF rats, feeding an 0.75% adenine diet, were randomly received intraperitoneal injection of 3AB (10mg/kg/d) or vehicle once a day. Serum levels of blood urea nitrogen (BUN), creatinine (Cr), calcium and phosphorus were measured by an autoanalyzer. Values are means ± S.D. (n=10-12 per group). ***P*＜0.01 vs the age-matched control.

**Supplementary Table 2.** **Serum biochemical parameters and body weight of CRF rats with PARP1 deficiency.**

| Parameters | control | CRF | CRF+Scr shRNA | CRF+PARP1 shRNA |
| --- | --- | --- | --- | --- |
| Body weight (g) | 446.25±42.52 | 264.23±25.64^**^ | 258.69±27.61 | 259.66±32.15 |
| Phosphorus(mM) | 2.49±0.21 | 4.59±0.37^**^ | 4.57±0.31 | 4.29±0.44 |
| Calcium (mM) | 2.29±0.24 | 2.51±0.26 | 2.46±0.28 | 2.53±0.29 |
| BUN (mM) | 7.41±1.02 | 81.25±6.42^**^ | 78.65±8.41 | 81.56±7.21 |
| Cr(µM) | 66.25±9.12 | 311.25±39.46^**^ | 300.58±42.56 | 306.52±44.86 |

Rat abdominal aortas were inoculated with adenovirus encoding Scrambled (Scr shRNA) or PARP1 shRNA at three weeks after the adenine diet, and then fed for three weeks. Serum levels of blood urea nitrogen (BUN), creatinine (Cr), calcium and phosphorus were measured by an autoanalyzer. Values are means ± S.D. (n=10-12 per group). ***P*＜0.01 vs the age-matched control.

**Supplementary Table 3**. **Serum biochemical parameters and body weight of CRF rats with PARP1 overexpression.**

| Parameters | control | CRF | CRF+Ad-Null | CRF+Ad-PARP1 |
| --- | --- | --- | --- | --- |
| Body weight (g) | 459.32±45.12 | 251.23±24.36^**^ | 249.68±29.49 | 264.35±32.98 |
| Phosphorus(mM) | 2.37±0.27 | 4.49±0.39^**^ | 4.48±0.58 | 4.39±0.59 |
| Calcium (mM) | 2.31±0.21 | 2.46±0.24 | 2.44±0.27 | 2.49±0.22 |
| BUN (mM) | 6.98±1.42 | 75.68±9.21^**^ | 82.12±6.66 | 80.24±6.75 |
| Cr(µM) | 69.12±7.25 | 302.12±37.56^**^ | 300.86±41.03 | 298.65±35.26 |

Rat abdominal aortas were inoculated with Ad-Null or Ad-PARP1 at three weeks after the adenine diet, and then fed for three weeks. Serum levels of blood urea nitrogen (BUN), creatinine (Cr), calcium and phosphorus were measured by an autoanalyzer. Values are means ± S.D. (n=10-12 per group). ***P*＜0.01 vs the age-matched control.

**Supplementary Table 4**. **Serum biochemical parameters and body weight of CRF rats under Runx2 deficiency.**

| Parameters | Control | CRF | CRF+Ad-Null+Scr shRNA | CRF+Ad-PARP1+Scr shRNA | CRF+Ad-Null+Runx2 shRNA | CRF+Ad-PARP1+Runx2 shRNA |
| --- | --- | --- | --- | --- | --- | --- |
| Body weight (g) | 477.58±59.22 | 268.95±31.25^**^ | 261.98±26.45 | 261.32±25.98 | 256.98±27.49 | 261.44±32.56 |
| Phosphorus(mM) | 2.41±0.29 | 4.51±0.28^**^ | 4.51±0.41 | 4.44±0.49 | 4.50±0.21 | 4.32±0.41 |
| Calcium (mM) | 2.33±0.22 | 2.49±0.33 | 2.51±0.24 | 2.45±0.19 | 2.44±0.28 | 2.46±0.27 |
| BUN (mM) | 6.58±1.75 | 78.88±7.52^**^ | 79.52±6.88 | 76.98±8.56 | 80.24±7.56 | 80.21±6.85 |
| Cr(µM) | 72.85±14.26 | 311.25±41.25^**^ | 320.45±36.98 | 299.65±37.54 | 298.64±39.64 | 294.65±41.32 |

Rat abdominal aortas were inoculated with Ad-Scr shRNA, or Ad-Runx2 shRNA together with Ad-Null or Ad-PARP1 at three weeks after the adenine diet. Six weeks later, the weights and blood samples were collected. Serum levels of blood urea nitrogen (BUN), creatinine (Cr), calcium and phosphorus were measured by an autoanalyzer. Values are means ± S.D. (n=10-12 per group). ***P*＜0.01 vs the age-matched control.

**Supplementary Table 5. Serum biochemical parameters and body weight of CRF rats under miR-204 manipulation.**

| Parameters | Control | CRF | CRF+Ad-Null | CRF+Ad-PARP1 | CRF+Ad-PARP1+control agomiR | CRF+Ad-PARP1+agomiR-204 |
| --- | --- | --- | --- | --- | --- | --- |
| Body weight (g) | 449.88±58.98 | 268.23±31.43^**^ | 259.15±39.56 | 257.17±28.23 | 261.53±26.57 | 248.83±14.37 |
| Phosphorus(mM) | 2.44±0.21 | 4.55±0.28^**^ | 4.67±0.29 | 4.73±0.33 | 4.48±0.41 | 4.46±0.23 |
| Calcium (mM) | 2.29±0.18 | 2.47±0.11 | 2.44±0.19 | 2.46±0.12 | 2.40±0.11 | 2.47±0.09 |
| BUN (mM) | 6.51±1.39 | 86.92±5.31^**^ | 81.62±7.56 | 81.07±9.56 | 82.54±8.56 | 82.03±6.74 |
| Cr(µM) | 74.22±7.65 | 322.67±52.47^**^ | 315.24±45.69 | 320.67±40.04 | 316.54±34.26 | 293.16±33.23 |

Rat abdominal arteries were inoculated with Ad-Null or Ad-PARP1 at three weeks after the adenine diet, and miR-204 agomir was intravenously injected through the tail vein for three consecutive days. Serum levels of blood urea nitrogen (BUN), creatinine (Cr), calcium and phosphorus were measured by an autoanalyzer. Values are means ± S.D. (n=10-12 per group). ***P*＜0.01 vs the age-matched control.
