# Supplementary material for: Poly(ADP-ribose) polymerase 1 accelerates vascular calcification by upregulating Runx2
Source: Nat Commun. 2019 Mar 13;10:1203. doi: 10.1038/s41467-019-09174-1 (PMC6416341; doi:10.1038/s41467-019-09174-1)
Supplement: Supplementary file 3 — Reporting Summary [file 41467_2019_9174_MOESM3_ESM.pdf]

nature research

Corresponding author(s): Kai Huang

Last updated by author(s): Feb 25, 2019

Reporting Summary

Nature Research wishes to improve the reproducibility of the work that we publish. This form provides structure for consistency and transparency in reporting. For further information on Nature Research policies, see Authors & Referees and the Editorial Policy Checklist.

Statistics

For all statistical analyses, confirm that the following items are present in the figure legend, table legend, main text, or Methods section.

n/a	Confirmed	
☐	☒	The exact sample size (*n*) for each experimental group/condition, given as a discrete number and unit of measurement
☐	☒	A statement on whether measurements were taken from distinct samples or whether the same sample was measured repeatedly
☐	☒	The statistical test(s) used AND whether they are one- or two-sided<br>*Only common tests should be described solely by name; describe more complex techniques in the Methods section.*
☐	☒	A description of all covariates tested
☐	☒	A description of any assumptions or corrections, such as tests of normality and adjustment for multiple comparisons
☐	☒	A full description of the statistical parameters including central tendency (e.g. means) or other basic estimates (e.g. regression coefficient) AND variation (e.g. standard deviation) or associated estimates of uncertainty (e.g. confidence intervals)
☐	☒	For null hypothesis testing, the test statistic (e.g. *F*, *t*, *r*) with confidence intervals, effect sizes, degrees of freedom and *P* value noted<br>*Give P values as exact values whenever suitable.*
☒	☐	For Bayesian analysis, information on the choice of priors and Markov chain Monte Carlo settings
☒	☐	For hierarchical and complex designs, identification of the appropriate level for tests and full reporting of outcomes
☒	☐	Estimates of effect sizes (e.g. Cohen's *d*, Pearson's *r*), indicating how they were calculated

Our web collection on statistics for biologists contains articles on many of the points above.

Software and code

Policy information about availability of computer code

Data collection	Image Lab, Stepone Software v2.3, cellSens Dimension.
Data analysis	Microsoft Excel, ImageJ (JACoP v.2.0), SPSS software (version 22.0, SPSS Inc), Adobe Photoshop CC2015, Adobe Ilustrator CC 2015, Microsoft Powerpoint.

For manuscripts utilizing custom algorithms or software that are central to the research but not yet described in published literature, software must be made available to editors/reviewers. We strongly encourage code deposition in a community repository (e.g. GitHub). See the Nature Research guidelines for submitting code & software for further information.

Data

Policy information about availability of data

All manuscripts must include a data availability statement. This statement should provide the following information, where applicable:

- Accession codes, unique identifiers, or web links for publicly available datasets
- A list of figures that have associated raw data
- A description of any restrictions on data availability

The data that support the findings of this study are available within the article and its Supplementary Information or from the corresponding authors on reasonable request. The source data underlying Figs 4a, 4e, 5d, 5i, 6c, and 6i and Supplementary Figs 5b, 5d, 6b and 7a are provided as a Source Data file

Field-specific reporting

Please select the one below that is the best fit for your research. If you are not sure, read the appropriate sections before making your selection.

☒ Life sciences ☐ Behavioural & social sciences ☐ Ecological, evolutionary & environmental sciences

For a reference copy of the document with all sections, see nature.com/documents/nr-reporting-summary-flat.pdf

Life sciences study design

All studies must disclose on these points even when the disclosure is negative.

Sample size	The sample size for each experiment, n, is included in the results section and the associated figure legend.
Data exclusions	No data were excluded from the analyses.
Replication	Data are representatives of at least three independent experiments with similar results and plotted as mean ± SD
Randomization	Mice were random allocated into experimental group
Blinding	The investigators were blinded to group allocation during data collection and analysis.

Reporting for specific materials, systems and methods

We require information from authors about some types of materials, experimental systems and methods used in many studies. Here, indicate whether each material, system or method listed is relevant to your study. If you are not sure if a list item applies to your research, read the appropriate section before selecting a response.

Materials & experimental systems

n/a	Involved in the study	
☐	☒	Antibodies
☐	☒	Eukaryotic cell lines
☒	☐	Palaeontology
☐	☒	Animals and other organisms
☒	☐	Human research participants
☒	☐	Clinical data

Methods

n/a	Involved in the study	
☒	☐	ChIP-seq
☒	☐	Flow cytometry
☒	☐	MRI-based neuroimaging

Antibodies

Antibodies used	Antibody against PAR (Cat#4335-MC-100; 1:1000 for WB; 1:100 for IF) were purchased from Trevigen (MD, USA). Antibodies against PARP1 (Cat#9532; 1:1000), Runx2 (Cat#12556; 1:1000 for WB; 1:100 for IF), pSTAT3 (Cat#9145; 1:1000), STAT3 (Cat#12640; 1:1000), pJAK2 (Cat#3776; 1:1000), JAK2 (Cat#3230; 1:1000), pSRC (Cat#6943; 1:1000) and SRC (Cat#2109; 1:1000) were obtained from Cell Signaling Technology (MA, USA). Antibodies against SMA (Cat#ab8226; 1:2000 for WB; 1:100 for IF), osteopotin (Cat#ab8448; 1:500), OC (Cat#ab13420; 1:500), osterix (Cat#ab209484; 1:1000), MSX2 (Cat#ab223692; 1:1000). Sox9 (Cat#ab185966; 1:1000), GAPDH (Cat#ab8245; 1:2000) and Argonaute-2 (Cat#ab32381;1:100) were purchased from Abcam (Cambridge, UK). Antibodies against SM22 (Cat#10493-1-AP; 1:1000) and SMMHC (Cat#21404-1-AP; 1:1000) were obtained from Proteintech (IL, USA). Antibodies against Pit1 (Cat#a4117; 1:500) and Pit2 (Cat#a6739; 1:500) were purchased from Abclonal (Wuhan, China). The Col1A1 (Cat#NB600-408; 1:500) antibody was purchased from – Novus (CO, USA). TRPM3 (Cat#GTX16612; 1:500) antibody was purchased from GeneTex (CA, USA).
Validation	All antibodies are commercial available and validated.

Eukaryotic cell lines

Policy information about cell lines

Cell line source(s)	A7r5 cell is from the aorta of rat. HASMC cell is from the aorta of human.
Authentication	The A7r5 and HASMC cell lines were obtained from ATCC.
Mycoplasma contamination	All cell lines are negative for mycoplasma contamination

Commonly misidentified lines (See ICLAC register)	N/A

Animals and other organisms

Policy information about studies involving animals; ARRIVE guidelines recommended for reporting animal research

Laboratory animals	eight-week-old male Wistar rats
Wild animals	N/A
Field-collected samples	N/A
Ethics oversight	All experimental protocols were approved by the Ethics Committee of Tongji Medical College, Huazhong University of Science and Technology, and were performed in accordance with relevant institutional and national guidelines and regulations.

Note that full information on the approval of the study protocol must also be provided in the manuscript.
